# Epigenetics in diabetic nephropathy, immunity and metabolism

**DOI:** 10.1007/s00125-017-4490-1

**Published:** 2017-11-11

**Authors:** Samuel T. Keating, Janna A. van Diepen, Niels P. Riksen, Assam El-Osta

**Affiliations:** 1 Department of Internal Medicine, Department of Internal Medicine (463), Radboud University Medical Center, Nijmegen, PO Box 9101, 6500 HB Nijmegen, the Netherlands; 2 Central Clinical School, Monash University, 99 Commercial Road, Melbourne, VIC 3004 Australia; 3 Department of Pathology, The University of Melbourne, Parkville, VIC Australia; 4 Hong Kong Institute of Diabetes and Obesity, Prince of Wales Hospital, The Chinese University of Hong Kong, Hong Kong, SAR China

**Keywords:** Chromatin, Diabetes, Diabetic complications, Diabetic nephropathy, Epigenetics, EWAS, Histone, Innate immune memory, Vascular

## Abstract

When it comes to the epigenome, there is a fine line between clarity and confusion—walk that line and you will discover another fascinating level of transcription control. With the genetic code representing the cornerstone of rules for information that is encoded to proteins somewhere above the genome level there is a set of rules by which chemical information is also read. These epigenetic modifications show a different side of the genetic code that is diverse and regulated, hence modifying genetic transcription transiently, ranging from short- to long-term alterations. While this complexity brings exquisite control it also poses a formidable challenge to efforts to decode mechanisms underlying complex disease. Recent technological and computational advances have improved unbiased acquisition of epigenomic patterns to improve our understanding of the complex chromatin landscape. Key to resolving distinct chromatin signatures of diabetic complications is the identification of the true physiological targets of regulatory proteins, such as reader proteins that recognise, writer proteins that deposit and eraser proteins that remove specific chemical moieties. But how might a diverse group of proteins regulate the diabetic landscape from an epigenomic perspective? Drawing from an ever-expanding compendium of experimental and clinical studies, this review details the current state-of-play and provides a perspective of chromatin-dependent mechanisms implicated in diabetic complications, with a special focus on diabetic nephropathy. We hypothesise a codified signature of the diabetic epigenome and provide examples of prime candidates for chemical modification. As for the pharmacological control of epigenetic marks, we explore future strategies to expedite and refine the search for clinically relevant discoveries. We also consider the challenges associated with therapeutic strategies targeting epigenetic pathways.

**Electronic supplementary material:**

The online version of this article (10.1007/s00125-017-4490-1) contains a slideset of the figures for download, which is available to authorised users.

## Introduction

Vascular disease affecting nearly all types of blood vessels is common to both type 1 and type 2 diabetes mellitus. Accelerated rates of clinically defined macrovascular complications, such as myocardial infarction and stroke, which result from large vessel atherosclerosis remain the leading causes of morbidity and premature mortality in the diabetic population. Diabetes is also associated with the occurrence of adverse microvascular complications, manifesting clinically as retinopathy, neuropathy and nephropathy.

Extensive debate surrounds the extent to which diabetic microvascular and macrovascular complications represent a continuous pathological spectrum. Closely related to this debate is the question of why not all people with diabetes complications experience more advanced forms of vascular disease. Though yet to be completely defined mechanistically, the persistent and harmful effects of antecedent hyperglycaemia may at least partly explain the variation in vascular deterioration. Nonetheless, the fundamental reasons why a proportion of diabetic individuals appear to be protected from serious complications remain poorly understood. Despite the promises of the genetic revolution, contemporary knowledge of the impact of genetic variation on diabetes does not adequately explain the disproportionate distribution and severity of diabetic vascular complications.

Realisation of novel preventative and therapeutic approaches hinges on improved characterisation of the molecular events and interactions that underlie the development and progression of diabetic vasculopathology. Interestingly, however, some in the field have shifted their research focus to understanding the post-translational and covalent chemical chromatin modifications that contribute to transcriptional regulation via structural adaptation. Insight from cultured cells and preclinical models, as well as clinical samples, has highlighted the importance of chromatin modifications in the persistent inflammatory response to glycaemic variability. Thus, epigenetics may be able to provide an explanation as to why some individuals with diabetes are predisposed to developing vascular disease and are more likely to progress to advanced stages of complications and/or develop other associated vascular pathologies.

## Glycaemic memories and vascular complications of diabetes

### The problem of hyperglycaemic persistence

As mentioned above, long-term inadequate glycaemic control is a major risk factor in the development of vascular complications. Despite the proclivity for patients with good metabolic control to have a significantly decreased risk for developing complications, vascular disease may still develop and progress even with intensive treatment regimens [1]. This is particularly true for individuals with a history of suboptimal glycaemic control who develop vascular disease despite good current metabolic control, a phenomenon known as ‘glycaemic memory’ or ‘legacy effect’.

The landmark Diabetes Control and Complications Trial (DCCT) was the first to demonstrate that achieving near-normal blood glucose levels ameliorates microvascular complications of type 1 diabetes [2]. Moreover, by switching both groups of study participants to the intensive insulin regimen in the wake of the successful completion of DCCT, the Epidemiology of Diabetes Interventions and Complications (EDIC) follow-up study not only confirmed the durability of the effects of glucose control on more advanced stages of complications, but also revealed that, despite stringent long-term glycaemic control, previous periods of suboptimal blood glucose continued to be a risk factor for chronic microvascular complications [3]. While provocative, these findings were not without precedent, as vascular memory of prior hyperglycaemia had been suggested by earlier studies of various experimental animal models [4–6]. As regards type 2 diabetes, observational studies suggest the enduring consequences of antecedent hyperglycaemia underlying vascular risk [7–9].

### Microvascular and macrovascular complications

Although hyperglycaemia is demonstrably a principal cause of microvasculopathy—the microvasculature of the retina is particularly susceptible to excess glucose and diabetic nephropathy is not observed in the absence of hyperglycaemia [10]—the efficacy of glucose-lowering interventions to reduce cardiovascular risk is still questioned [11]. Are the pathogenic characteristics of microvascular disease also related to the development of macrovascular disease? Indeed, diabetic individuals with microvascular complications are especially prone to accelerated atherosclerosis and premature mortality [12]. Of the numerous organ systems affected by diabetes, the impact on renal function is the most pronounced. The diabetic kidney is considered a primary failing organ and its clinical features are increasingly considered to be indicative of overall vascular damage. Individuals with diabetic nephropathy endure an exceptionally high risk of cardiovascular disease, and both increased urinary albumin excretion and reduced GFR are prognostic of cardiovascular morbidity and mortality [13].

### Diabetes in the GWAS era

Unlike the near inexorable progression to retinopathy, more than half of all individuals with type 1 diabetes do not develop renal complications [14]. While insufficient metabolic and haemodynamic control, as well as prolonged disease duration, may explain some cases, the fact that individuals with strict compliance can develop clinically evident nephropathy whereas many individuals with similar or worse control do not, illustrates the disproportionate distribution of the diabetic nephropathy burden [10]. Similarly, not all people with microalbuminuria progress to macroalbuminuria or end-stage renal disease (ESRD), apparently protected despite decades of chronic hyperglycaemia and haemodynamic stress. Furthermore, increased risk of renal disease aggregates in families [15], as exemplified by the finding that the incidence of nephropathy in diabetic children of individuals with diabetic nephropathy is more than three times that in children of individuals without renal disease [16, 17].

Thus, the search for genetic factors associated with diabetic nephropathy susceptibility, using initially linkage analyses, candidate gene-based approaches, and, more recently, hypothesis-free GWAS has been extensive. Yet, even with the advent of modern sequencing technologies, intensive efforts have yielded only a limited number of consistent genetic associations, and the impact on clinical management has so far been negligible [18]. Large collaborations drawing from sufficiently powered sampling, such as those recently published by the Surrogate markers for Micro-and Macro- vascular hard endpoints for Innovative diabetes Tools (SUMMIT) consortium [19], provide additional motivation for genetic studies in pursuit of the enigmatic heritability of chronic kidney disease.

### Editing and interpreting chromatin modifications

Methylation is unique in the way that it is enriched at cytosine bases of the DNA template, primarily, but not exclusively, at cytosine–guanine (CpG) dinucleotides, as well as on the tails of chromatinised histones. When written by DNA methyltransferase enzymes (DNMT1, DNMT3a, and DNMT3b in humans) to the 5-carbon position of cytosine (5-methylcytosine, 5mC), the methyl modification is historically associated with transcriptional silencing by recruitment of specific factors that actively remodel the chromatin structure, as well as by the disruption of transcription factor binding sites. On the other hand, 5mC enrichment can preclude binding of transcriptional repressors such as CCCTC-binding factor (CTCF), which is associated with altered chromatin structures and thus aberrant gene activation [20]. Indeed, the precise location of the modification relative to genetically encoded regulatory elements is central to the epigenetic function of 5mC. Recent characterisation of the ten-eleven-translocation (TET) family of proteins that hydroxylate 5mC to 5-hydroxymethylcytosine (5hmC) has inspired strong interest in DNA demethylation pathways [21]. As for the role of the demethylation and chromatin remodelling by the TET proteins, the enrichment of 5hmC at the gene body is implicated in transcriptional activation. Indeed, transcriptional induction of adipocyte differentiation is dynamically regulated by the binding of CTCF to chromatin [22].

**Fig. 1:**
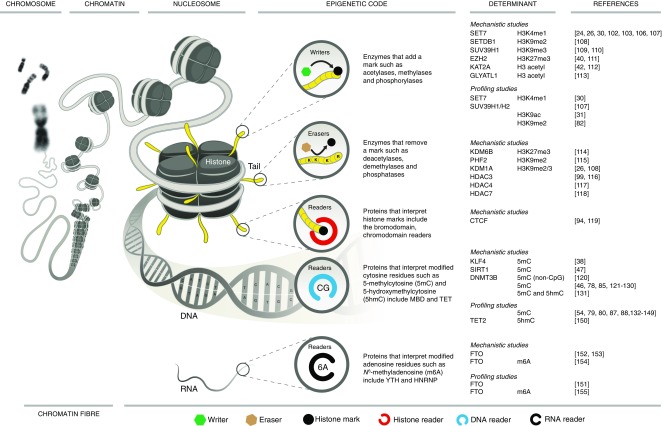
Codified signature of the diabetic epigenome. Readers, writers and erasers in diabetes. Modification of the diabetic epigenome includes post-translational modifications to the tails of histones, carried out by histone-modifying enzymes (known a ‘writers’), such as SET7 [24, 26, 30, 102, 103, 106, 107], SETDB1 [108], SUV39H1 [109, 110], EZH2 [40, 111], KAT2A [42, 112] and GLYATL1 [113]. Experimental studies that provide mechanistic insights for specific determinants are grouped to include the enzyme and corresponding modified histone, whereas informative profiling studies using clinical cohorts are separated with examples such as SET7 [30], SUV39H1/H2 [107], H3K9 acetylation [31] and H3K9me2 [82]. The epigenetic code is dynamic and eraser enzymes are implicated in diabetes such as KDM6B [114], PHF2 [115], KDM1A [26, 108], HDAC3 [99, 116], HDAC4 [117] and HDAC7 [118]. Protein readers such as CTCF recognise post-translational histone modifications including methylation of cytosine residues in CpG dinucleotides [94, 119]. Genome readers regulate transcriptional responses and include KLF4 [38], SIRT1 [47], as well as non-CpG methylation by DNMT3B [120]. The DNA template is subject to modification and recent experimental studies have shown an association with 5mC [46, 78, 85, 121–130] and 5hmC [131]. Clinical profiling studies for DNA modification have also shown an association with 5mC [54, 79, 80, 87, 88, 132–149] and 5hmC [150]. Post-transcriptional gene regulation by RNA modifications include the writers, erasers and readers of *N* ^6^-methyladenosine (m6A). FTO [151–153] is an m6A eraser implicated in metabolic homeostasis [154] and is associated with type 2 diabetes [155] CARM1, coactivator associated arginine methyltransferase 1; DNMT3B, DNA methyltransferase 3B; FTO, fat mass and obesity-associated protein; GLYATL1, glycine-*N*-acyltransferase like 1; HNRNP, heterogeneous nuclear ribonucleoprotein; KAT2A, K (lysine) acetyltransferase 2A; KDM, lysine (K)-specific demethylase; NSD, nuclear receptor binding SET domain protein; MBD, methyl-CpG binding domain protein; PHF2, PHD finger protein 2; PRMT, protein arginine methyltransferase; SETDB, SET domain bifurcated; SETMAR, SET domain and mariner transposase fusion protein; SIRT1, sirtuin 1; SUV39H, suppressor of variegation 3-9 homologue; YTH, YTH domain protein. Blank fields in the mechanistic and profiling studies refer to either enzymes or modified determinants that were not reported in the studies listed

Methylation of lysine and arginine residues on histone tails is similarly associated with both transcriptional activation and repression, depending not only on the position of the substrate residue within a specified histone tail, but also the degree of modification (mono-, di-, or tri-methylation, Table 1). Trimethylated lysines at position 4 of the H3 histone tail (H3K4m3) are associated with active promoters, whereas histones methylated at H3K9 and H3K27 are predominantly enriched at repressed genes. Monomethylated H3K4 (H3K4m1) denotes distal enhancers [23], and plays a regulatory role at specific promoters [24, 25]. A dynamic network of highly specific methyl writers (methyltransferase) and erasers (demethylase) regulate these and many other sites predominantly on the tails of H3 and H4 histones [26]. Such epigenetic marks are read by multi-subunit chromatin remodelling complexes, though their precise function, including interaction with traditional transcription factors, as well as mechanisms regulating gene-specific enrichment, remain to be definitively characterised (Fig. 1).

**Table 1:** Sites and regulators of chromatin modifications

Substrate	Target	Modification	Relationship to transcription	Writer
DNA	CpG	Cytosine methylation	Repressive/activating	DNMT1, DNMT3a, DNMT3b
H3 histone	H3R2	Arginine methylation	Repressive	PRMT6, CARM1
	H3K4	Lysine methylation	Activating	KMT2A-E, SET7, SETD3, SETMAR, SETD1A, SETD1B, NSD3, SMYD1, SMYD2, SMYD3
	H3R8	Arginine methylation	Repressive	PRMT5
	H3K9	Lysine acetylation	Activating	ELP3, KAT2A
	H3K14	Lysine methylation	Repressive	KAT2A, EHMT2, EZH2, SETDB1, SETDB2, SUV39H1, SUV39H2
		Lysine acetylation	Activating	CLOCK, KAT6A, KAT2A, MGEA5, KAT2B, KAT5
	H3R17	Arginine methylation	Activating	CARM1
	H3K18	Lysine acetylation	Activating	CREBBP, ELP3, EP300
	H3K23	Lysine acetylation	Activating	KAT2A, EP300
	H3R26	Arginine methylation	Activating	CARM1
	H3K27	Lysine acetylation	Activating	CREBBP, EP300
		Lysine methylation	Repressive	EZH1, EZH2, SETDB1, SETDB2, SUV39H1, SUV39H2, EHMT2, NSD3
	H3K36	Lysine methylation	Activating	SETD2, SETD3, SMYD2, SETMAR, NSD2
	H3K79	Lysine methylation	Activating	DOT1L
H4 histone	H4R3	Lysine methylation	Repressive/activating	PRMT1, PRMT7
	H4K5	Lysine acetylation	Activating	CREBBP, KAT2A, KAT5, KAT7, EP300
	H4K8	Lysine acetylation	Activating	KAT5, CREBBP, KAT2A, EP300, KAT7
	H4K12	Lysine acetylation	Activating	CREBBP, KAT2A, KAT5, EP300, KAT7
	H4K16	Lysine acetylation	Activating	CREBBP, KAT2A, EP300
	H4K20	Lysine methylation	Repressive	KMT5B, KMT5C, SET8

CARM1, coactivator associated arginine methyltransferase 1; CLOCK, clock circadian regulator; CREBBP, CREB binding protein; DNMT, DNA methyltransferase; DOT1L, DOT1 like histone lysine methyltransferase; EHMT2, euchromatin histone lysine methyltransferase 2; ELP3, elongator acetyltransferase complex subunit 3; EP300, E1A binding protein p300; EZH1, enhancer of zeste polycomb repressive complex 1 subunit; KAT, K (lysine) acetyltransferase; KMT, lysine (K)-specific methyltransferase; MGEA5, meningioma expressed antigen 5 (hyaluronidase); NSD, nuclear receptor binding SET domain protein; PRMT, protein arginine methyltransferase; SET7, SET domain containing lysine methyltransferase; SETD, SET domain containing; SETDB, SET domain bifurcated; SETMAR, SET domain and mariner transposase fusion protein; SMYD, SET and MYND domain containing; SUV39H, suppressor of variegation 3-9 homologue

Further emphasising the importance of chromatinised lysine residues is histone acetylation, which promotes an open chromatin structure by electrostatic charge disruption and facilitates the assembly of transcriptional machinery [27]. The acetyl-writing activities of histone acetyltransferases (HATs) are mechanistically opposed by histone deacetylases (HDACs), a dichotomy that has been exploited for the clinical treatment of heart disease and cancer [28, 29]. Furthermore, there is obvious interplay between histone methylation and acetylation in their competition for lysine substrates. Other important though less well studied histone modifications include phosphorylation, sumoylation, ubiquitination, ADP-ribosylation, and *O*-GlcNAcylation.

## Epigenetic changes in diabetic nephropathy

We have previously described the important role of the SET7 lysine methyltransferase in vascular endothelial cells in writing a specific high-glucose-mediated H3K4m1 signature at the promoter of the *RELA* gene [24, 26], which encodes the proinflammatory p65 subunit of NFκB. In accordance with the concept of glycaemic memory, this modification persisted in cultured human vascular cells and rodents beyond euglycaemic restoration. Importantly, the particular SET7-dependent H3K4m1 signature was recently identified in monocytes of diabetic individuals [30]. In addition, specific enrichment of histone acetylation was observed at several genes related to diabetes complications in DCCT/EDIC participants who received conventional treatment as compared with those who received intensive therapy [31], which may have implications for metabolic memory in vascular complications. Could similar mechanisms be responsible for the persistence and progression of diabetic nephropathy? Chromatin modifications are increasingly implicated in renal pathophysiology, and while the persistence of the majority of associations remains untested, it is evident that their influence reaches beyond roles in metabolic memory. We recently described the importance of cell-specific epigenetic changes in atherosclerosis [32] and in the section below we adopt a similar approach for diabetic nephropathy, highlighting recent key examples of chromatinised changes in podocytes and proximal epithelial cells.

### Transcriptional control in podocytes

Podocytes are visceral epithelial cells that line the urinary space of the renal corpuscle. These highly specialised cells derive their name from long interdigitating foot processes that form intercellular clefts called slit pores, bridged by diaphragms consisting of podocyte-specific proteins, such as NPHS1 (also known as nephrin) and NPHS2 (also known as podocin). Structural disturbance of the slit diaphragm proteins results in insufficient filtration and proteinuria, exemplified by congenital kidney failure arising from defects in nephrin [33]. Podocyte injury and loss, through detachment, apoptosis, or epithelial to mesenchymal transition (EMT) [34], are strong predictors of diabetic nephropathy progression [35] and are closely linked to glomerulosclerosis [36].

Expression of the pluripotency-associated Kruppel-like factor 4 (*KLF4*) [37] correlates positively with *NPHS1* expression and inversely with proteinuria in rodent and human podocytes [38]. Moreover, transient restoration of KLF4 in diseased glomeruli re-establishes the normal podocyte phenotype and attenuates proteinuria. KLF4 binds a specific motif on the *NPHS1* promoter and upregulates gene expression by reduced methylation or demethylation of adjacent CpG sites. Methyl profiling of cultured human podocytes overexpressing *KLF4* revealed widespread changes in 5mC, highlighted by reduced CpG methylation at promoters of other epithelial genes, such as *NPHS2* and *SYNPO* (which encodes synaptopodin), in striking contrast to hypermethylation at promoters of mesenchymal genes, such as *VIM* (which encodes vimentin) and *CTGF* (which encodes connective tissue growth factor), thereby indicating KLF4-dependent determination of podocyte phenotype by gene-specific methylation. The potential implications of these findings are significant, not only for slit diaphragm maintenance but also for epithelial to mesenchymal transition observed in advanced diabetic nephropathy. The slit diaphragm proteins NPHS1 and nephrin-like 3 (NEPH3) are encoded by genes (located on chromosome 19q13.12 in a head-to-head orientation) that share a bidirectional promoter and have been shown to be dependent on 5mC for co-regulation and expression [39].

Specifically within the diabetic setting, the chromatin-dependent regulation of glucose-mediated oxidative stress in podocytes is emerging as a critical mediator of diabetic nephropathy. For example, the H3K27-methylating function of enhancer of zeste 2 repressive complex 2 subunit (EZH2) recently emerged as an important regulator of diabetic nephropathy by repressing the transcription factor PAX6 and subsequently dampening expression of the endogenous antioxidant inhibitor thioredoxin-interacting protein (*TXNIP*). Inhibition of EZH2 augments proteinuria, podocytopathy, glomerular *Txnip* expression, and renal oxidative stress in a rat model of diabetes [40]. Disease-related genes are rarely regulated by a single mechanism, but, rather, can reflect the interplay of multiple epigenetic determinants. Indeed, glucose-mediated *Txnip* expression is coordinated by histone acetylation and methylation in kidneys from diabetic Sur1-E1506K+/+ mice [41]. Similarly, promoter CpG hypomethylation and concomitant H3 histone hyperacetylation by the GCN5 histone acetyltransferase were found to drive protein C-dependent expression of the critical mediator of oxidative stress p66^Shc^ in podocytes exposed to high glucose [42]. This epigenetic signature closely mirrors the specific hyperglycaemia-induced changes that activate and maintain p66^Shc^ expression beyond restoration of euglycaemia in cultured vascular endothelial cells and diabetic mice [43], therefore representing a possible epigenetic mechanism of glycaemic memory in the podocyte. The importance of H3 acetylation for p66^Shc^ gene regulation was further underscored by pharmacological and molecular experiments targeting class III HDACs, and specifically the NAD^+^-dependent SIRT1 HDAC [44].

### Transcriptional control in proximal tubule epithelial and glomerular mesangial cells

Contiguous with podocytes in renal structure, proximal tubular epithelial cells (PTECs) play a crucial role in renal function, reabsorbing much of the glucose and amino acids, as well as sodium, from the glomerular filtrate. Both high glucose and abnormal protein trafficking through the glomerulus induce inflammation and tubulointerstitial lesions through PTEC activation, and the extent of interstitial fibrosis ultimately determines the rate of decline in renal function [45]. Recent in vivo investigations highlight differential 5mC patterns associated with genes such as *Sglt2* (also known as *Slc5a2*) and *G6pc*, indicating that 5mC underlies selective glucose handling by PTECs in the kidney [46]. While PTECs isolated from *db/db* mice exhibited a comparable genome-wide methylation profile to PTECs from control animals, significant differences in 5mC were observed at genes implicated in sugar reabsorption (*Slc5a2*), nephropathy (*Met*) and hypertension (*Agt*) [46]. An important component of the renin-angiotensin system, *Agt* was shown to accumulate aberrant epigenetic changes early in the pathogenesis of diabetic nephropathy. Acetylated H3K9 was enriched at the *Agt* promoter as early as 5 weeks in the diabetic kidney and preceded both DNA hypomethylation and H3K9m3. The importance of H3 acetylation was further underscored by *Agt* transcriptional activation in response to HDAC inhibition. In contrast, these epigenetic changes were resistant to the glucose-lowering drug pioglitazone. In human diabetic nephropathy, microdissected tubuli from individuals with diabetic kidney disease exhibited differential methylation of genes implicated in fibrogenesis [47]. Central to nephron function is the network of capillaries that form the glomerulus. Activation of mesangial cells by advanced glycation end-products and high glucose increase proinflammatory and profibrotic cytokines, including angiotensin II (ANGII). Recent studies have shown that the ANGII type 1 receptor antagonist (AT1R) losartan, which is used to treat renal complications of diabetes, alters post-translational modifications on histones in glomeruli from *db*/*db* mice [48]. In mesangial cells cultured under hyperglycaemic conditions, losartan attenuates histone acetylation at *RAGE* (also known as *AGER*), *PAI1* (also known as *SERPINE1*) and *MCP-1* (also known as *CCL2*) promoters.

### Chromatin modifications regulate inflammation in diabetic nephropathy

Kidney biopsies from experimental diabetes models or individuals with diabetes are characterised by enhanced macrophage infiltration [49, 50]. Furthermore, a localised proinflammatory response is well characterised in the vasculature and kidneys under diabetic conditions, exemplified by proinflammatory cytokine and chemokine secretion and the overproduction of reactive oxygen species [51, 52]. The local presence and contribution of activated macrophages to the aetiology of both diabetic nephropathy and cardiovascular disease [53] may point towards a general systemic role in diabetes complications. Studies have identified a potential role of chromatin modifications in monocyte-derived inflammatory gene expression in the context of diabetes, including persistent vascular complications [30, 31, 54]. Long-term memory spanning decades in vascular cells such as macrophages could potentially be explained by persistent epigenetic profiles of progenitor cells, though such an association is yet to be demonstrated. In the acute setting, chromatin modifications in monocytes have been implicated recently in functional changes associated with the exciting concept of trained innate immunity (also termed innate immune memory). This is a fascinating new field with tremendous scope and biological implications, not only with respect to inflammatory diseases, but for all diseases in which monocytes play a pathological role [55].

#### Trained immunity: chromatin-dependent memory

Immunological memory had classically been viewed as being characteristic of only the adaptive immune system (T and B lymphocytes). However, it has recently emerged that innate immune cells exhibit memory-like behaviour, characterised by an increased proinflammatory response to secondary infections [55–57]. Importantly, the trained memory of monocytes is non-specific, meaning that an encounter with a certain pathogen can also protect against infection by unrelated pathogens [56, 57]. The heightened responsiveness of monocytes is characterised by enhanced secretion of proinflammatory mediators associated with widespread changes in chromatin patterns [58]. Burgeoning interest is rapidly uncovering an intricate program of chromatin modification underlying not only training for a heightened immune response, but also tolerance to re-stimulation, which is basically the opposite of training [59]. Some aspects of this macrophage memory are at least partly dependent on the induction of latent enhancers [60], constitutively unmarked distal elements that acquire signature epigenetic features of enhancers (H3K4m1 and H3K27ac) upon stimulation with certain microbial products. Moreover, after the initial stimulation, H3K4m1 persists at decommissioned regulatory elements to mediate a faster response to re-stimulation, further emphasising the role of this specific histone modification in transcriptional memory and inflammation [24].

The non-specific trained memory of monocytes is thought to have beneficial effects in numerous immunological settings, including vaccination programmes [61]. In contrast, trained immunity may play a maladaptive role in chronic inflammatory (metabolic) diseases such as atherosclerosis [62, 63]. Especially since microbial training of monocytes not only enhances responsiveness to subsequent pathogens, but also primes transcription of chemokines and scavenger receptors to promote foam cell formation [62, 64]. This suggests an important link between trained immunity and metabolic diseases. Even more relevant are experiments demonstrating that non-pathogen-related pro-atherosclerotic metabolites such as oxidised LDL (oxLDL) and lipoprotein(a) [Lp(a)] can also induce trained immunity [64, 65]. Monocytes trained by oxLDL or Lp(a) exhibit a long-term pro-atherogenic monocyte phenotype, which is associated with specific and persistent H3K4m3 enrichment at activated promoters, and is accordingly attenuated by pan-methyltransferase inhibition [64, 65].

Chronic hyperglycaemia associates with monocyte activation, induced directly by glucose or by other endogenous compounds associated with hyperglycaemia, such as AGEs [66, 67]. Whether glucose or AGEs promote epigenetic reprogramming of monocytes, macrophages or progenitor cells remains to be determined; however, this could play an important role in the phenomenon of hyperglycaemic memory in individuals with diabetes [68].

#### Immunometabolism and diabetic vascular complications

The epigenetic reprogramming of monocytes clearly involves marked changes in cellular metabolism, which is determined by their activation status [58, 69]. Specifically, oxidative phosphorylation is used as a primary metabolic process by resting cells, which contrasts with a profound switch to aerobic glycolysis (Warburg effect) upon activation [69]. The glycolytic switch is under the control of the Akt–mammalian target of rapamycin (mTOR)– hypoxia-inducible factor-1α (HIF1α) pathway and aimed at optimising immune cell function, including macromolecular synthesis and enhanced cytokine production. Recent multilevel -omics analysis revealed that glycolysis, glutaminolysis and cholesterol synthesis are non-redundant pathways for the induction of trained immunity by the microbial cell wall component β-glucan [70].

Interestingly, the metabolic state of the cell is linked to a particular epigenetic program [71]. Indeed, the intracellular changes in the metabolic milieu may in fact drive the epigenetic reprogramming of monocytes during trained immunity [72]. For example, accumulation of fumarate in β-glucan-trained cells, due to glutamine replenishment of the tricarboxylic acid cycle, integrates immune and metabolic circuits with epigenetic regulation by inhibiting the lysine demethylase 5 (KDM5) histone demethylase. Moreover, fumarate induces epigenetic reprogramming similar to β-glucan-mediated trained immunity [70].

The relationship between metabolic processes and epigenetic changes has so far been studied predominantly in the field of cancer research [73], but could potentially have major implications for cellular behaviour in disturbed metabolic environments such as diabetes [74]. Assuming that chronic hyperglycaemia increases glucose availability as a substrate for innate immune cells, this may affect intracellular metabolism (e.g. stimulate glycolysis), including changes in intermediate metabolites that promote epigenetic changes [68]. Chromatin-dependent immunological training by fumarate [70] is particularly relevant for inflammation in diabetic nephropathy because this metabolite was recently shown to accumulate in the kidneys of diabetic rats [75].

### Predisposition, progression and prognosis

The heritability of epigenomic signatures continues to be intensely debated, though several plausible mechanisms of transgenerational transmission of acquired phenotypes have been described (reviewed elsewhere [76]), including metabolic traits [77]. Recent studies demonstrate that epigenetic regulation underlying phenotypic determinants of adult metabolic health is influenced in utero and by the early postnatal environment [74, 78]. The potential for shared environmental exposures to impart similar epigenetic patterns among related individuals cannot discount the influence of genetic variation. Perhaps the phenotypic consequences of some susceptible genes are only revealed under specific patterns of epi-regulation induced by environmental variation or diabetes-specific processes, thereby confounding their discovery by traditional GWAS. Previously unmarked genomic regulatory elements can be commissioned by stimulus-dependent persistent chromatin modifications [60], and similar undiscovered mechanisms could be responsible for a latent susceptibility to diabetic nephropathy or exacerbate the effects of disease-associated genetic variants.

Expansion of high-throughput sequencing technologies to include the profiling of chromatin modifications on a genome-wide scale represents a new approach towards understanding predisposition to diabetic nephropathy. Studies comparing diabetic individuals with and without diabetic nephropathy reported differential DNA methylation at numerous genes, including several previously identified by GWAS [54, 79, 80]. One example is the gene encoding unc-13 homologue B (*UNC13B*), which is associated with glucose-mediated apoptosis in glomerular cells [81], and is hypermethylated near its transcription start site in peripheral blood cells of type 2 diabetes patients with diabetic nephropathy [79]. Furthermore blood derived from individuals with type 1 diabetes revealed an association between diabetic nephropathy and differential methylation at genes involved in mitochondrial function [80]. This study compared methylation patterns in African-American and Hispanic diabetic individuals with ESRD and diabetic people without nephropathy. Interestingly, participants with ESRD being treated with haemodialysis show significantly reduced methylation.

Immune cell subtypes are distinguished by epigenetic profiles [82], and differences between monocytes of different individuals are relatively stable [83], consistent with monocytes being an appropriate and practical source of material. Such a profiling strategy provides opportunities to gain greater insight into gene-regulating events specific to the pathological properties of monocytes in diabetes complications, as well as other vascular cell types. Epigenomes derived from peripheral blood [79] and saliva [80] exhibit marked differences between individuals with and without diabetic nephropathy, indicating, at least in principle, the utility of such a proxy for disease risk. However, the importance of cell-specific epigenomes cannot be overlooked [32].

While certainly an attractive approach to understanding complex disease phenotypes, several challenges limit the interpretability of epigenomic profiles. Notably, the aforementioned studies have used methylation array hybridisation technology that predominantly assays promoters, and to date there are no unbiased profiles of DNA methylation in the context of diabetic nephropathy. Genome coverage is critical to understand differential methylation outside promoter regions. For example, the application of next-generation sequencing generates comprehensive maps of DNA methylation data, thereby reducing the limitations often attributed to array composition [54]. Contrary to popular belief as it specifically pertains to the field of oncology, not all methylation sites are born equal [84]. While it is appreciated that genes are repressed by methylation, repression is not strictly restricted to promoters of genes and highlights the importance of methodological detection [85].

#### Shaping the diabetes epigenome

Altered DNA methylation patterns at specific loci can distinguish phenotypic cases from controls to reveal possible causal mechanisms. However, the cross-sectional approach can also be confounded by reverse causation, where the interrogated epigenomes are shaped by (rather than cause) the disease [86], as described recently in studies of BMI [87] and type 1 diabetes [88]. Exerting an even greater influence is the DNA sequence itself, which is estimated to account for up to 80% of inter-individual DNA methylation [86]. To circumvent both issues of causality, epigenome profiling strategies should be reconsidered to include concurrent genotyping and transcriptome profiling of the same cells from a single individual [86].

Inter-individual epigenetic differences may prove to be valuable predictive biomarkers of diabetic nephropathy susceptibility and development. Progression of atherosclerosis can indeed be associated with the degree of DNA methylation within plaques [89], and prospective approaches could provide similar insight into the role of chromatin modifications in diabetic nephropathy, with potential prognostic applications. Sampling of individuals prior and subsequent to disease onset permits the discovery of epigenetic changes that precede and possibly even predict the overt phenotype, while reducing the effects of genetic variation that confound cross-sectional studies [86]. Further strengthening the relationship between epigenetics and metabolic memory, recent longitudinal profiling of monocyte DNA methylation from the same individuals at 7–6 year intervals identified loci-specific differential DNA methylation established during the DCCT that persists for several years during the EDIC Study. A noteworthy discovery was the persistent hypomethylation of nephropathy-associated *TXNIP*, an effect that was replicated in a cell culture model of hyperglycaemic variability [54].

**Fig. 2:**
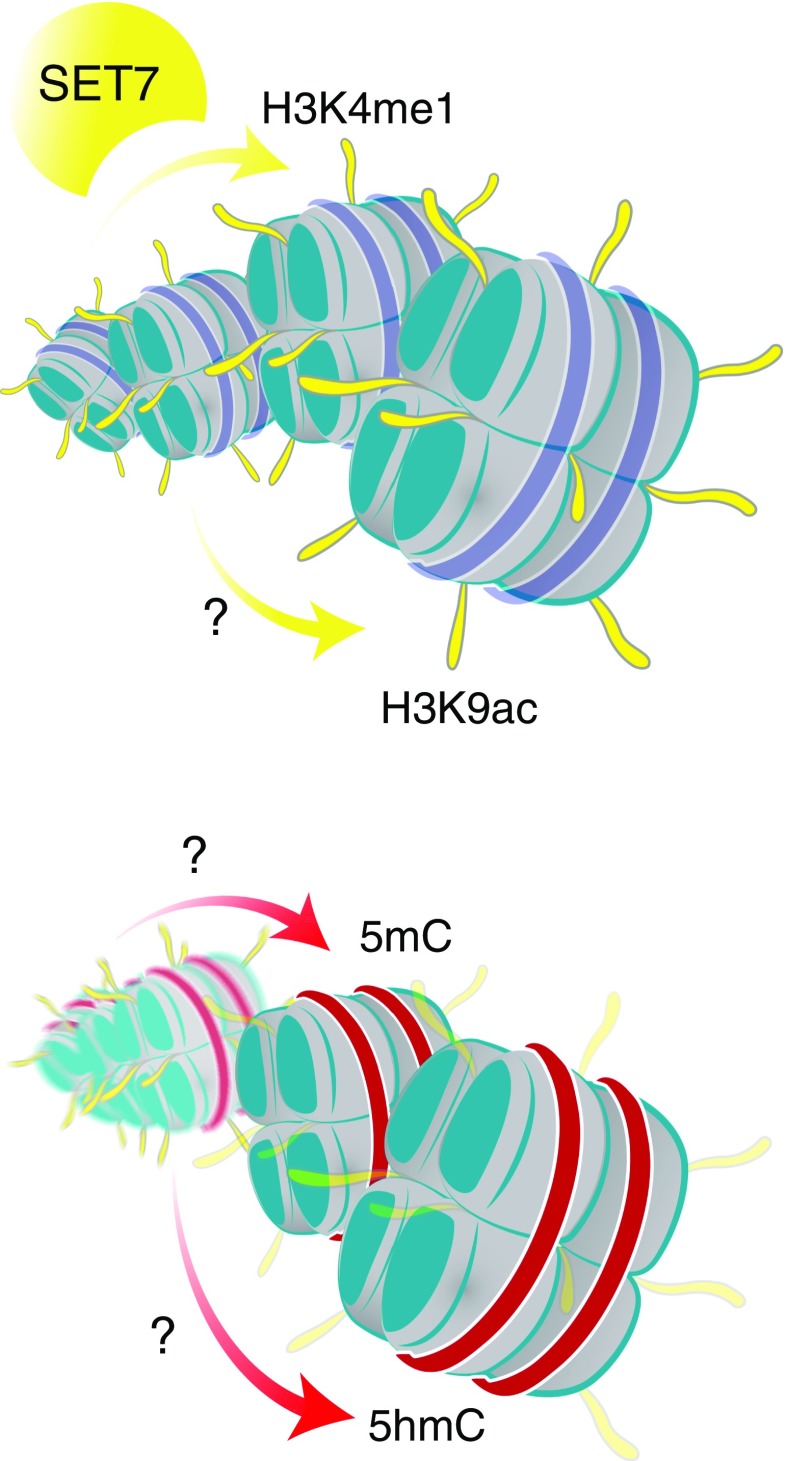
Epigenetic and metabolic memory nexus. Persistent epigenetic changes in the context of transient, medium- and long-term metabolic memory. In certain cell types, hyperglycaemic memory exists. For example, in response to hyperglycaemia, SET7 methyltransferase enzyme writes mono-methylation of histone H3 lysine 4 (H3K4me1) in vascular endothelial cells, and this methylation is retained over the long term. The effectiveness of transient hyperglycaemic stimuli to tightly control the expression of target genes implicated in vascular dysfunction and inflammation relies on ROS-mediated pathways. Indeed, a hyperglycaemic sensor (SET7) and the transfer of a chemical group (mono-methylation on H3K4) to a gene are two common principles implicated in metabolic memory. These regulatory events serve to remodel chromatin to precisely decorate target genes such as the p65 subunit of NFkB, with H3K4me1 corresponding with persistent transcriptional activation [24, 26, 30, 102]. The long-term contribution of hyperglycaemic signalling cues derived from the monocytes of type 1 diabetic individuals from the DCCT and EDIC trials show HbA_1c_ levels and H3K9ac are also tightly linked [31]. The control of genes related to the NFkB inflammatory pathway by modifications exhibits common principles of epigenetic control that serves as a paradigm for metabolic memory. The mechanism conferring H3K9ac and metabolic memory remains poorly characterised. Clearly, this explains only part of the epigenetic complexity, in the metabolic memory example and using monocytes derived from the DCCT and EDIC trials, genomic modification, specifically, 5mC is also an important determinant in the control of gene expression [54]. The mechanism underlying 5mC modification in metabolic memory remains poorly characterised. Recent advances in our understanding of the mechanisms of genomic modification show 5hmC, a DNA base derived from 5mC by oxidation by TET enzymes, is implicated in transposon activity associated with exposure to adverse in utero programming and gestational diabetes [150]. These findings emphasise that cytosine residue modification plays an important role in the regulation of genes implicated in diabetes

As discussed previously, 5mC is a powerful regulatory determinant, and given that 60–90% of the cytosines in the adult vertebrate cell contain CpG methylation, the prospect that many other functional sites within the genome are potentially altered would seem highly likely, although there is limited experimental evidence of this (Fig. 2). The most direct mechanism by which CpG methylation could alter gene expression would be the prevention or enhancement of the binding of transcriptional machinery at ubiquitous consensus binding sites. For example, CTCF is a chromatin insulator that serves to regulate access of distant enhancers to promoters [90]. Indeed, CTCF binding is methylation sensitive [91], binding imprinted control regions only at the unmethylated parental allele to regulate specific gene expression patterns [92]. CTCF binding also protects regions from DNA methylation [93]. This diversity in methylation distribution provides a direct mechanism to regulate gene expression, offering a simple and elegant means of controlling key target genes in signalling complexes and core pathways implicated in type 2 diabetes [94]. The alternative possibility is that transcriptional repressors that assemble on chromatin recognise the methylation moiety together with other transcriptional components [95]. Indeed, methylation-specific binding proteins potentially serve to control gene expression of many functional sites within the genome in a chromatin context, thereby offering an attractive model to explain the capacity of gene function more effectively [96].

## Targeting chromatin modifications in the clinic

With increased exploration of chromatin modifications in various medical contexts, it may be possible in the future not only to track causal mechanisms of vascular disease, but also to capture an individual patient’s position within a complex spectrum of pathophysiological processes, thereby supporting tailored approaches to the anticipation and prevention of diabetic complications [97]. The epigenome is responsive to internal and external stimuli as diverse as disease-specific processes, nutrition [74] and exercise [98]. Exploitation of this plasticity has fast become a novel avenue of investigation to improve dysregulated gene function. Whereas metabolic manipulation of the chromatin landscape is a relatively recent suggestion, pharmacological compounds that modulate epigenetic regulators have a longer history in the clinic. Common to both strategies is the imperative challenge of specificity.

The emerging picture of epigenetic regulation is one of remarkable complexity. Defining the tissue-specific relative contributions of epigenetic writers and erasers remains an unresolved but critical issue for developing novel therapeutic epigenetic modulators. Whereas HDAC3 deletion from the macrophage is vasculo-protective [99], deletion of the same enzyme from endothelial cells exacerbates macrovascular disease [100]. Many enzymes that modify histones also target specific amino acids on other proteins, including transcription factors, to post-translationally regulate their stability and activity, with major implications for the interpretation of gene expression profiles. In addition to writing H3K4m1, SET7 methylates a variety of transcription factors in different cellular contexts [101], and is therefore implicated in chromatin-dependent and chromatin-independent gene regulation [102, 103]. Furthermore, evidence is emerging to suggest that histone-modifying enzymes can post-translationally modify and regulate each other [74], meaning that a single enzyme could potentially influence many distinct modifications. Finally, the mechanisms driving loci-specific enrichment of chromatin modifications remain largely uncharacterized, though several examples of non-coding RNA and transcription factor co-recruitment have recently emerged [104, 105]. The respective repertoire of gene-localising mechanisms is likely to reflect cell type specificity of distinct gene programs.

Despite these formidable challenges, the epigenome is rich in opportunity. Compounds that target epigenetic pathways are increasingly investigated pre-clinically, and drugs that are already used in clinical management of diabetes may impact the epigenetic landscape. For example, metformin prevents trained immunity by the Bacillus Calmette–Guérin (BCG) vaccine via mTOR inhibition and suppression of glycolysis [69]. Whether this holds true for immunological training in the context of diabetes remains to be elucidated.

## Conclusion

Increasingly accessible technologies that permit unbiased acquisition of genome-wide patterns boast the capacity to transform our understanding of the chromatin landscape in the occurrence and progression of complex diseases. The immense potential for epigenetics to explain many aspects of diabetic vascular complications is evident in recent scientific literature. By sensitising the genome to environmental variation, these molecular signatures shape diverse phenotypes and functional programs. Chromatin modifications influence deleterious changes in gene expression that, under some circumstances, can endure improvements in metabolic management. Similarly, persistent epigenetic modifications drive the non-specific memory of proinflammatory macrophages, a process that is increasingly implicated in vascular disease and could prove to be instrumental to resolving the important debate as to whether diabetic microvascular and macrovascular pathology share a common pathology. Epigenomic profiling of circulating cells may further shed light on the phenotypic variation and disproportionate burden of diabetic vascular complications. Combined with classical genetic approaches, epigenomic profiling has potential to identify molecular trajectories underlying diabetic vascular disease development. While the extent that pathological chromatin changes can be manipulated in human diabetic complications remains to be established, the clinical applicability of epigenetic interventions will be greatly advanced by a deeper understanding of the cell type-specific functions and interactions of chromatin-modifying machinery in the diabetic vasculature.

## Electronic supplementary material

ESM Downloadable slideset (PPTX 771 kb)

## Abbreviations

5hmC: 5-Hydroxymethylcytosine
5mC: 5-Methylcytosine
BCG: Bacillus Calmette–Guérin vaccine
CpG: Cytosine–guanine dinucleotide
CTCF: CCCTC-binding factor
DCCT: Diabetes Control and Complications Trial
DNMT: DNA methyltransferase
EDIC: Epidemiology of Diabetes Interventions and Complications
ESRD: End-stage renal disease
EZH2: Enhancer of zeste 2 repressive complex 2 subunit
HAT: Histone acetyltransferase
HDAC: Histone deacetylase
KLF4: Kruppel-like factor 4
mTOR: Mammalian target of rapamycin
oxLDL: Oxidised low-density lipoprotein
Lp(a): Lipoprotein(a)
NPHS1: Nephrin
PTEC: Proximal tubular epithelial cell
TET: Ten-eleven translocation
TXNIP: Thioredoxin-interacting protein
