# Supplementary material for: Epigenetics in diabetic nephropathy, immunity and metabolism
Source: Diabetologia. 2017 Nov 11;61(1):6–20. doi: 10.1007/s00125-017-4490-1 (PMC6448927; doi:10.1007/s00125-017-4490-1)
Supplement: Supplementary file 1 — (PPTX 771 kb) [file 125_2017_4490_MOESM1_ESM.pptx]

## Slide 1
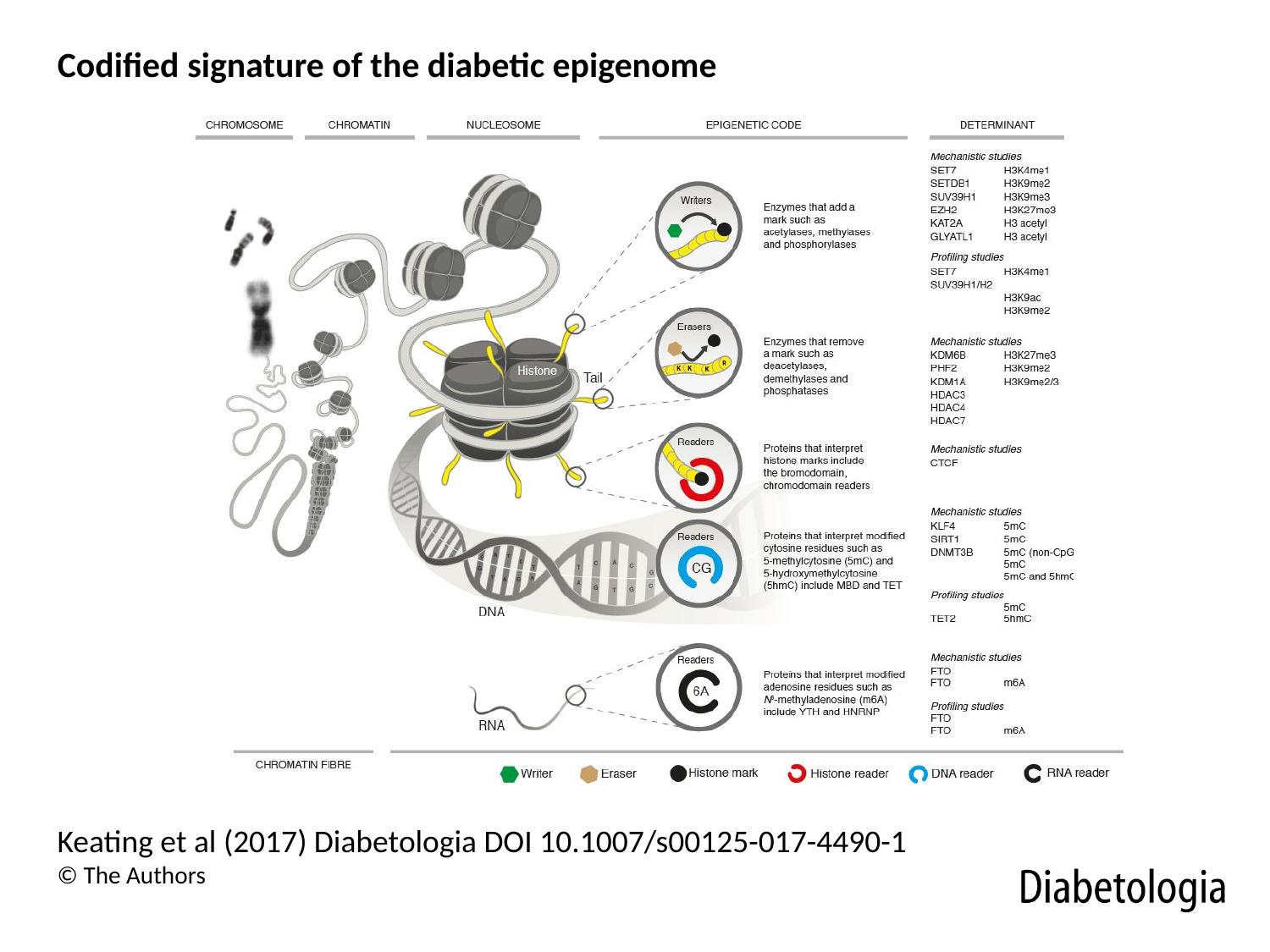

Codified signature of the diabetic epigenome
FIGURE HERE
Keating et al (2017) Diabetologia DOI 10.1007/s00125-017-4490-1
© The Authors

## Slide 2
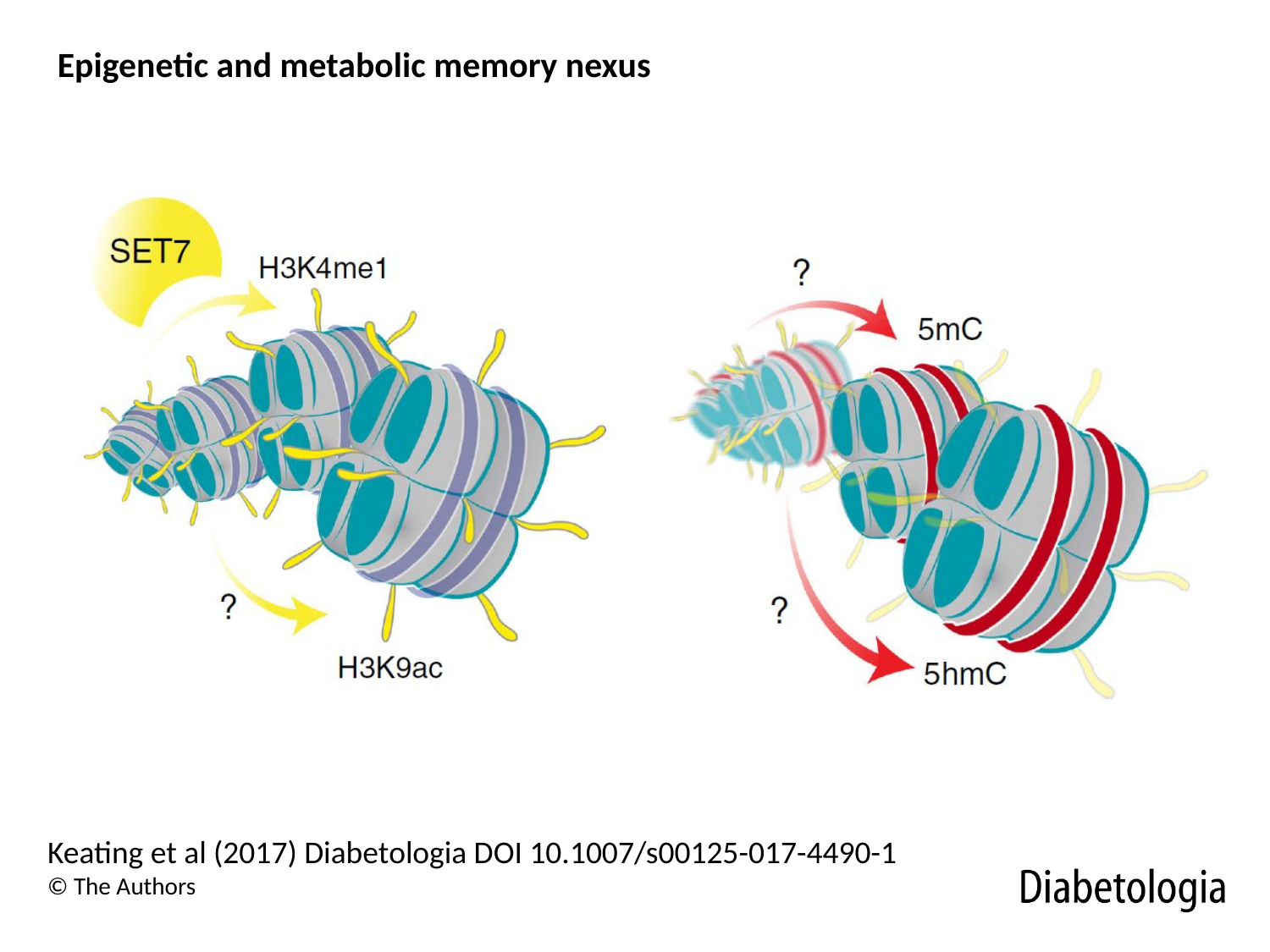

Epigenetic and metabolic memory nexus
Keating et al (2017) Diabetologia DOI 10.1007/s00125-017-4490-1
© The Authors
